# Hypoxic preconditioning improves intraoperative oxygenation and postoperative recovery in children undergoing thoracoscopic surgery: a randomized controlled trial

**DOI:** 10.3389/fmed.2026.1801428

**Published:** 2026-05-07

**Authors:** Ting Xiao, Lei Wang, Dong Jie Pei, Shui Bing Zhang, Jing Hua Wang, Guang Xian Yang, Shuang Quan Qu

**Affiliations:** 1 Department of Anesthesiology, The Affiliated Children’s Hospital of Xiangya School of Medicine, Central South University (Hunan Children’s Hospital), Changsha, Hunan, China; 2 Department of Thoracic Surgery, The Affiliated Children’s Hospital of Xiangya School of Medicine, Central South University (Hunan Children’s Hospital), Changsha, Hunan, China

**Keywords:** hypoxic preconditioning, lung protection, one-lung ventilation, pediatric anesthesia, postoperative recovery, remote ischemic preconditioning, thoracoscopic surgery

## Abstract

**Background:**

Hypoxia preconditioning (HPC) and remote ischemic preconditioning (RIPC) are potential lung-protective strategies, but their efficacy in pediatric thoracic surgery remains unclear. This randomized controlled trial aimed to evaluate the effects of HPC, alone or in combination with RIPC, in children undergoing video-assisted thoracoscopic pulmonary resection.

**Methods:**

In a single-center, 2 × 2 factorial randomized trial, 160 children (<18 years) undergoing thoracoscopic lung surgery were allocated to four groups: Control (no preconditioning), HPC (three cycles of 5-min hypoxia/ventilation in the non-dependent lung), RIPC (three cycles of 5-min limb ischemia/reperfusion), and combined HPC + RIPC. The primary outcome was the PaO_2_/FiO_2_ ratio at 30 min of one-lung ventilation (OLV). Secondary outcomes included the duration of postoperative mechanical ventilation, hospital length of stay, and incidence of postoperative pulmonary complications (PPCs).

**Results:**

A total of 139 patients were included in the final analysis. Compared with the control group, HPC significantly improved oxygenation at 30 min of OLV (PaO_2_/FiO_2_: 256.4 ± 60.3 vs. 201.3 ± 65.0 mmHg; *p* = 0.022), reduced median mechanical ventilation duration (45 vs. 102.5 min; *p* < 0.001), and shortened hospital length of stay (7 vs. 11 days; *p* = 0.001). HPC also reduced the incidence of PPCs (53% vs. 81%; *p* = 0.021), with the largest reduction observed in pleural effusion. The addition of RIPC did not enhance these outcomes, and RIPC alone had no significant effect.

**Conclusion:**

HPC was associated with improved intraoperative oxygenation and enhanced postoperative recovery in children undergoing thoracoscopic pulmonary surgery. These findings suggest that HPC may represent a simple and promising lung-protective strategy in pediatric thoracic anesthesia.

**Clinical trial registration:**

Identifier ChiCTR2000038658, https://www.chictr.org.cn/showproj.html?proj=61845.

## 1 Introduction

Video-assisted thoracoscopic surgery (VATS) has become increasingly adopted in pediatric patients due to its minimal invasiveness and improved postoperative outcomes (1). However, the use of one-lung ventilation (OLV), a necessary component of VATS, may predispose pediatric patients to intraoperative hypoxemia, ventilation/perfusion (V/Q) mismatch, and postoperative pulmonary complications (PPCs) due to both mechanical and inflammatory lung injury (2).

Hypoxia preconditioning (HPC) is a physiological adaptation strategy that involves brief cycles of hypoxia followed by reoxygenation, enhancing tissue tolerance to subsequent insults (3–5). Experimental studies have shown that HPC mitigates lung injury in various models, including acute lung injury (ALI), acute respiratory distress syndrome (ARDS), and hyperoxia-induced damage (6–8). In adult clinical populations, HPC has been associated with improved oxygenation and reduced systemic inflammation during thoracic procedures involving OLV (9, 10). Despite these promising findings, the effectiveness of HPC in children undergoing thoracic surgery remains poorly defined.

Remote ischemic preconditioning (RIPC), another organ-protective strategy, involves transient ischemia/reperfusion in a remote tissue—typically a limb—to trigger systemic protective responses (11). While RIPC has demonstrated cardiopulmonary protective effects in pediatric cardiac surgery (12), recent trials have reported mixed or null results in neonatal populations (13), underscoring the context-dependent efficacy of this approach.

To date, limited randomized data exist regarding whether HPC or RIPC, alone or in combination, confers pulmonary protection in the pediatric thoracic surgical setting. Given the unique physiological characteristics of children, it remains unclear whether these preconditioning strategies can be extrapolated from adult or cardiac models to the thoracoscopic pediatric population.

To our knowledge, this study represents the first randomized evaluation of HPC and RIPC in children undergoing VATS. We hypothesized that these preconditioning strategies would attenuate lung injury, primarily manifested by improved intraoperative oxygenation [defined as the ratio of arterial oxygen partial pressure to fractional inspired oxygen (PaO_2_/FiO)] and accelerated postoperative clinical recovery.

## 2 Methods

This prospective, single-center, 2 × 2 factorial randomized controlled trial was conducted in accordance with the Consolidated Standards of Reporting Trials (CONSORT) guidelines. The protocol received approval from the Hunan Children’s Hospital Ethics Committee (HCHLL-2020-34; Chair Prof. ZH. X) on September 2, 2020, and was prospectively registered at the Chinese Clinical Trials Registry (ChiCTR2000038658; registration date: September 27, 2020). All experiments were conducted in accordance with the Declaration of Helsinki and relevant guidelines. Informed consent was obtained from all subjects and/or their legal guardians prior to enrollment.

### 2.1 Study population

The study population comprised children aged <18 years who were scheduled to undergo video-assisted thoracoscopic pulmonary surgery for congenital cystic adenomatoid malformation with an OLV duration of > 30 min. Informed consent was obtained from the parents of the participating children. Only the children whose parents provided informed consent were enrolled. Children with abnormal organ function, American Society of Anesthesiologists (ASA) stage IV disease, history of asthma, active upper airway or systemic infection, and limb trauma were excluded. In addition, children with unstable circulation during the perioperative period, OLV failure, and those who were lost to follow-up were also excluded.

### 2.2 Randomization and blinding

Using computer-generated permuted block randomization (block size 8), participants were stratified by age groups (<2 years vs. ≥ 2 years) and allocated to four groups (Control, HPC, RIPC, HPC + RIPC) via sequentially numbered opaque envelopes. The randomization sequence was generated by an independent statistician not involved in patient recruitment, intervention delivery, or outcome assessment.

Double-blinding was implemented as follows:

Patients/parents: provided standardized information about “perioperative respiratory interventions” without group-specific details to avoid expectancy bias.
Surgeons & outcome assessors: kept blinded to group allocations by a dedicated anesthesia team responsible for HPC/RIPC interventions. All surgical teams and clinical outcome evaluators (e.g., radiologists assessing postoperative lung complications) had no access to intervention details.
Data analysts: accessed only de-identified datasets with no links to group assignments.
Unblinded anesthesiologists: performed HPC/RIPC interventions behind visual barriers in the operating room to prevent unintentional signaling to the surgical team. Emergency unblinding protocols (e.g., for severe hypoxemia requiring protocol deviation) were predefined but not activated during the study.

### 2.3 Anesthesia protocol

All children enrolled in the study followed the ASA guidelines for fasting. Midazolam (0.1 mg/kg), sufentanil (0.3–0.5 ug/kg), cisatracurium (0.1 mg/kg,) and propofol (3 mg/kg) were used to induce anesthesia. Anesthesia was maintained with sevoflurane (1 vol%) and a continuous infusion of remifentanil (0.2–0.35 ug/kg/min) and dexmedetomidine (0.2 ug/kg/h). Arterial and central venous catheterizations were performed under ultrasound guidance. Additional doses of cisatracurium (0.1 mg/kg) were administered as needed to maintain the train-of-four (TOF) value. The bispectral index (BIS) was maintained between 45 and 55 by adjusting the dosage of remifentanil and dexmedetomidine. The children were transferred to the care unit without extubation postoperatively. The tracheal tube was not removed until the following extubation criteria were met: spontaneous breathing tidal volume (Vt) > 6 mL/kg, age-appropriate respiratory rate, hemodynamic stability (systolic blood pressure and heart rate within ±20% of baseline), peripheral oxygen saturation(SpO_2_) ≥ 95% (FiO₂ ≤ 40%), and exhaled carbon dioxide <50 mmHg. The chest drainage tube was removed when the chest radiography or computed tomography (CT) results were normal.

### 2.4 One lung ventilation

OLV was initiated using an Arndt endobronchial blocker (AEB) 5 Fr (Arndt Blocker; Cook Critical Care, IL, USA). Tracheal intubation was performed initially in patients aged > 2 years, and a bronchial blocker was introduced into the appropriate bronchus through the ports of a Y-shaped adapter under fiberoptic bronchoscopy (FOB) guidance. The smallest 5 Fr AEB was initially inserted into the trachea 2 cm past the vocal cords in patients aged < 2 years. A half-size smaller cuffed endotracheal tube (ETT) was inserted through the cords adjacent to the extraluminal blocker subsequently. The FOB was inserted through the ETT to facilitate blocker visualization and the manipulation of the selected bronchus.

Lung protection was achieved during OLV by maintaining a low Vt of 6 mL/kg and using positive end-expiratory pressure (PEEP of 5 cmH_2_O). FiO_2_ was set as 1.0 when the peripheral oxygen saturation (SpO_2_) fell below 92%, then slowly decreased. The lowest oxygen concentration was used to maintain a blood oxygen saturation of >95%. CPAP was administered to the non-ventilated lung if the SpO_2_ remained <92% after adjusting FiO_2_. Double-lung ventilation (DLV) was initiated temporarily if this approach failed. Lung recruitment for residual atelectasis was performed at the end of the surgery.

### 2.5 Hypoxic preconditioning

HPC was performed after confirming the correct placement of AEB and before making the incision. HPC (9) was induced by implementing three cycles of intermittent ventilation in the operative lung, comprising 5 min of OLV, followed by 5 min of DLV. The ventilation protocol entailed that Vt be maintained at 6 mL/kg during OLV and 8 mL/kg during DLV, based on the standard body weight of the children.

### 2.6 Remote ischemic preconditioning

RIPC was performed after confirming the position of the AEB and before making the incision. RIPC (14) was induced by implementing three cycles of 5 min of ischemia/reperfusion of the thigh, utilizing an arterial pressure cuff inflated to 30 mmHg higher than the systolic arterial pressure during the ischemic period, followed by 5 min of reperfusion with a deflated cuff.

### 2.7 Monitoring

Standard monitoring of blood pressure (BP), SpO_2_, invasive arterial blood pressure (IAP), heart rate (HR), and skin temperature was performed. Arterial blood gas parameters, including pH; PCO_2_; PaO_2_; and the glucose, lactate, and hemoglobin levels, were measured at the following time points: after introduction (T0), before OLV (T1), after 30 min of OLV (T2), at the end of OLV (T3), at the end of the surgery (T4), and 2 h post-surgery (T5).

The serum levels of interleukin-6 (IL-6), tumor necrosis factor-ɑ (TNF-ɑ), and malondialdehyde (MDA) were measured at all six time points. The IL-6, TNF-ɑ, and MDA levels were measured using an electro-chemiluminescence immunoassay (Elecsys, Roche, Mannheim, Germany), following standard hospital laboratory procedures. Blood samples were collected from the indwelling central venous lines using 1 cc serum separator tubes and centrifuged. Analysis of the blood samples was conducted immediately, or the samples were stored at −80 °C before analysis.

### 2.8 Primary and secondary outcome measures

The primary outcome was the PaO_2_/FiO_2_ ratio measured at 30 min after the initiation of OLV_,_ reflecting intraoperative gas exchange during thoracoscopic pulmonary surgery.

#### 2.8.1 Registry deviation statement

The trial was prospectively registered at the Chinese Clinical Trial Registry (ChiCTR2000038658). Although the initial trial registration listed several mechanistic biomarkers (NF-κB, IL-6, IL-10, and SOD, and MDA) as primary indicators, the study focus was prospectively refined during the conduct of the trial and prior to database lock and any outcome data analysis, during the prespecified statistical planning phase, to designate PaO_2_/FiO_2_ at 30 min of OLV as the primary clinical endpoint. This refinement was incorporated into the finalized statistical analysis plan and sample size calculation. However, the registry entry was not updated accordingly, representing an omission in registry synchronization. This deviation is explicitly reported here to ensure transparency.

Secondary outcomes included intraoperative respiratory parameters, postoperative mechanical ventilation duration, length of hospital stay, the incidence of PPCs and exploratory inflammatory and oxidative stress biomarkers. PPCs (15, 16) were defined as the occurrence of respiratory complications within 72 postoperative hours, including respiratory infection, respiratory failure, pleural effusion, atelectasis, pneumothorax, bronchospasm, or aspiration pneumonia.

### 2.9 Data collection

The demographic and clinical data, such as patient age, weight, ASA grade, surgical category, duration of surgery, and OLV, were recorded. In addition, the intraoperative cardiorespiratory parameters, such as the mean arterial pressure (MAP), HR, and SpO_2_, were recorded, in addition to the peak and standard monitoring parameters and arterial blood gas parameters. The displacement of AEB was also recorded at each study time point.

### 2.10 Statistical analysis

The sample size calculation was based on the PaO_2_/FiO_2_ ratio during OLV, serving as the definitive clinical endpoint. The sample size calculation and statistical analysis plan were finalized prior to database lock and unblinding. Although the initial registration referenced a preliminary sample size targeting biochemical endpoints, the final sample size was determined to ensure adequate statistical power for detecting clinically meaningful differences in oxygenation across the four randomized groups.

Based on preliminary data, the estimated mean PaO_2_/FiO_2_ values during OLV in the CR, HPC, RIPC, and HPC + RIPC groups were 201.3, 223, 208.7, and 260.3, respectively, with a standard deviation of 77.3. A total of 133 participants had to be enrolled to detect differences with a power of 0.8 and an alpha risk of 5%. Considering a dropout or ineligibility rate of 20%, 160 participants were enrolled.

All statistical analyses were conducted according to a modified intention-to-treat (mITT) principle and performed using SPSS (v25.0, SPSS Inc., Chicago, IL, USA). Patients were excluded post-randomization only when predefined conditions precluded valid assessment of the primary outcome, including failure of OLV, intraoperative hemodynamic instability requiring protocol deviation or withdrawal of consent.

The primary outcome was analyzed using a linear mixed-effects model consistent with a 2 × 2 factorial design. Fixed effects included HPC, RIPC, time, and the HPC × RIPC interaction, with a random intercept for each participant to account for within-subject correlation. Main effects and interaction effects were estimated with corresponding 95% confidence intervals.

Data distribution was assessed using the Shapiro–Wilk test. Continuous variables are presented as mean ± standard deviation (SD) for normally distributed data or median (interquartile range, IQR) for non-normally distributed data, as appropriate. Secondary continuous outcomes were performed using ANOVA or nonparametric tests (Kruskal–Wallis or Friedman tests), depending on data distribution. Categorical data are presented as counts and percentages and were analyzed using the chi-square test or Fisher’s exact test, as appropriate. *Post hoc* comparisons were adjusted for multiple testing using the Holm–Bonferroni method. Statistical significance was set at *p* < 0.05.

## 3 Results

### 3.1 Study population

**Figure 1:**
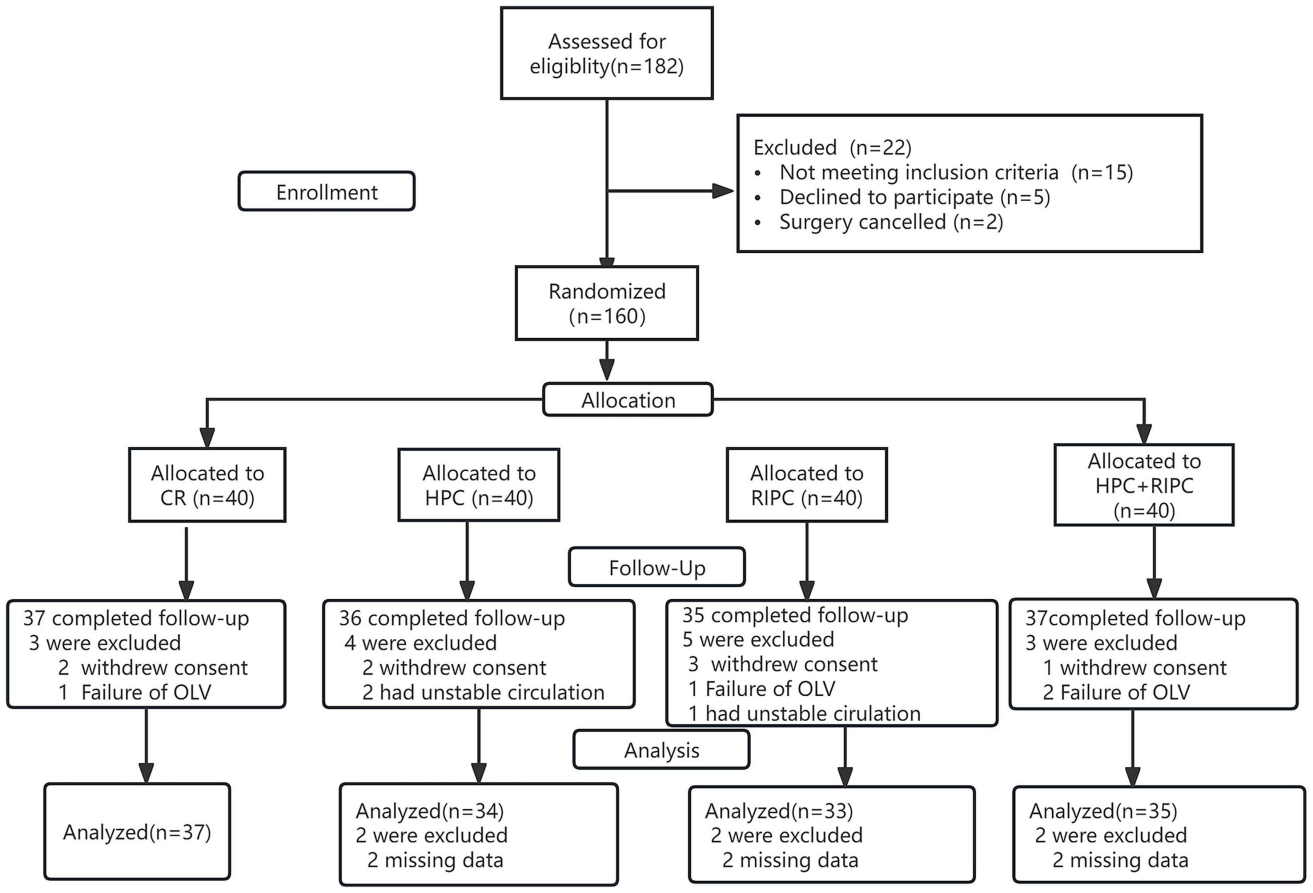
Study flow diagram. CR, Control; HPC, Hypoxia preconditioning; RIPC, Remote ischemic preconditioning; OLV, One-lung ventilation.

From September 2020 to February 2022, 182 children were assessed for eligibility. After excluding 22 cases (15 protocol violations, 5 parental declinations, 2 surgical cancellations), 160 patients underwent randomization. Following randomization, 21 patients met predefined post-randomization exclusion criteria and were excluded from the modified intention-to-treat (mITT) analysis, including withdrawal of consent (*n* = 8), missing primary outcome data (*n* = 6), failure of OLV (*n* = 4),and intraoperative hemodynamic instability requiring protocol deviation (*n* = 3). This yielded 139 analyzable patients (Control: 37; HPC: 34; RIPC: 33; HPC + RIPC: 35) (Figure 1). Importantly, this final sample size exceeded the minimum of 133 participants required to achieve 80% statistical power for the primary outcome. Groups were balanced in baseline characteristics (Table 1).

**Table 1:** The demographic data and surgical details.

Variable	CR (*n* = 37)	HPC (*n* = 34)	RIPC (*n* = 33)	HPC+RIPC (*n* = 35)	*p* value
Sex					0.661
Female	17 (46%)	14 (41%)	17 (52%)	13 (37%)	
Age (yr)	2.7 (0.9, 6.3)	3 (1.5, 7.4)	2.5 (0.6, 6.0)	2.5 (1.2, 6.7)	0.698
Weight (kg)	13.0 (8.3, 17.8)	13.5 (10.5, 23)	12.5 (7.5, 18.3)	11.9 (9.9, 22)	0.351
Height (cm)	92.5 (73, 110)	93 (77, 124)	89 (70, 115)	87 (77, 121)	0.558
BMI (kg/m2)	15.1 (14.4, 16.4)	15.4 (14.5, 18.6)	14.9 (14.0, 15.9)	15.6 (14.5, 17.9)	0.138
ASA score					0.351
I	2 (5%)	0	3 (9%)	3 (9%)	
II	35 (95%)	34 (100%)	30 (91%)	32 (91%)	
III	0	0	0	0	
Type of operation					0.931
Segmentectomy or wedge resection	26 (70%)	22 (65%)	25 (76%)	22 (63%)	
Bilobectomy	0	0	0	0	
Total operative time (min)	120 (75, 173.8)	119 (90, 170)	118 (73.5, 152)	137.5 (107.8, 193.8)	0.420
SLV time (min)	64.5 (41,107.3)	78 (44,125)	68 (38.5,101)	83 (63.8,123.8)	0.448
Input fluid volume (mL)	500 (335, 700)	450 (380, 650)	500 (325, 575)	500 (350, 705)	0.888
Intraoperative blood loss (mL)	22.5 (10, 72.5)	30 (10, 50)	20 (7.5, 40)	20 (8.8, 57.5)	0.377
Urine volume (mL)	80 (30, 200)	100 (55, 200)	70 (35, 125)	100 (80, 237.5)	0.209
Blood transfusion volume (mL)^a^	0 (0, 0)	0 (0, 0)	0 (0, 0)	0 (0, 0)	0.680

Data are presented as the count (percentage) and median (IQR), *p < 0.05 versus control group. BMI, Body mass index; SLV, Single lung ventilation; ASA, American Society of Anesthesiologists; CR, Control; HPC, Hypoxia preconditioning; RIPC, Remote ischemic preconditioning; Three cases in HPC required blood transfusion, with the patients receiving 160, 240, and 320 mL of blood. Four cases in RIPC required blood transfusion; two patients received 160 mL of blood, one patient received 150 mL of blood, and one patient received 80 mL of blood. Two patients in HPC+RIPC required blood transfusion, with the patients receiving 140 and 180 mL of blood.

a Five cases in CR required blood transfusion; three patients received 160 mL of blood, one patient received 220 mL of blood, and one patient received 480 mL of blood.

### 3.2 Primary outcome

**Figure 2:**
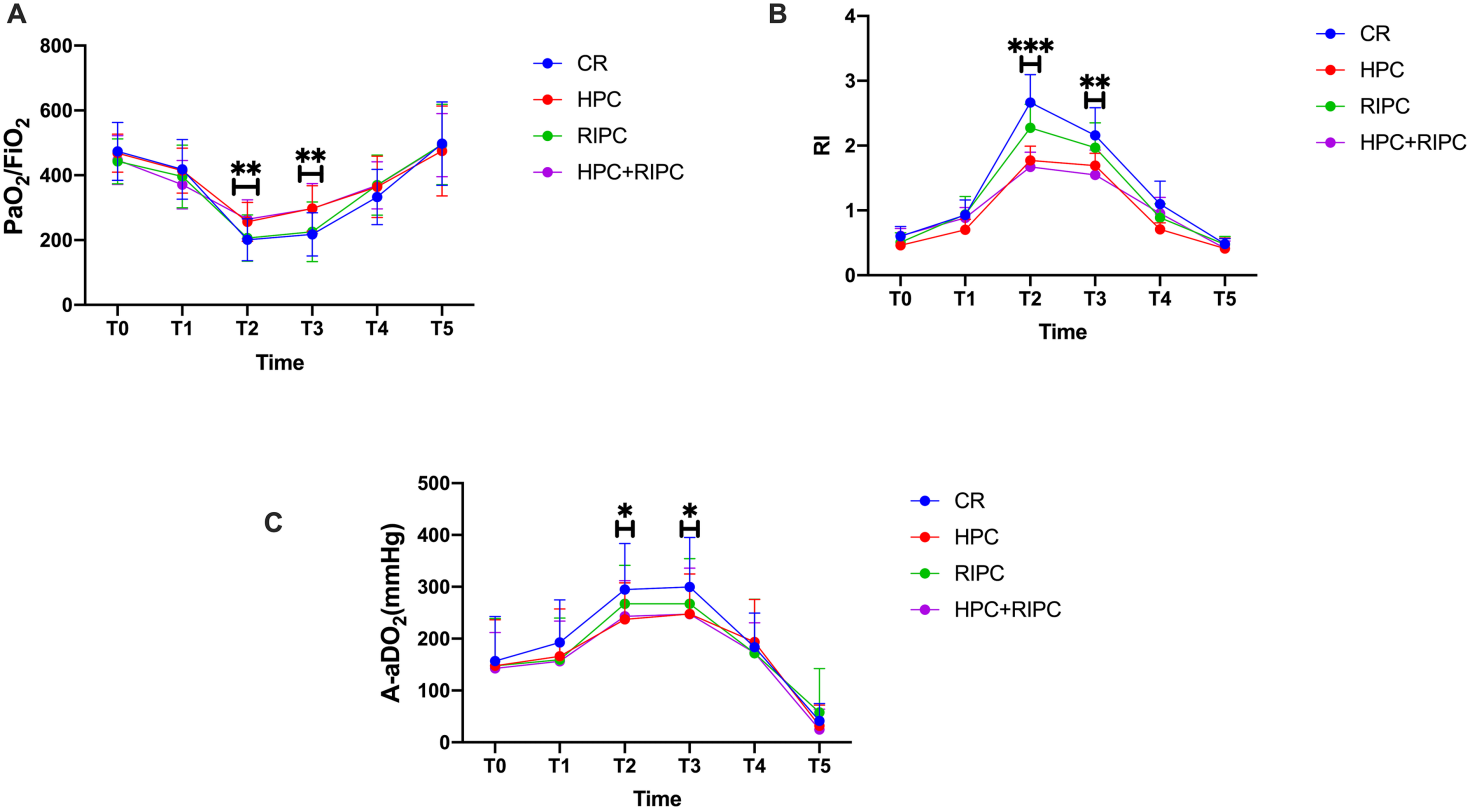
Effect of preconditioning on intraoperative oxygenation and respiratory mechanics. Changes in **(A)** PaO_2_/FiO_2_ ratio; **(B)** Respiratory index (RI); **(C)** Alveolar—arterial oxygen tension difference (A-aDO_2_) across six time points_._ Data are presented as mean ± SD. *p < 0.05, **p < 0.01, and ***p < 0.001 versus the Control group. Abbreviations: CR, Control; HPC, Hypoxia preconditioning; RIPC, Remote ischemic preconditioning; OLV, One-lung ventilation; T0, after introduction; T1, before OLV; T2, after 30 min of OLV; T3, at the end of OLV; T4, at the end of surgery; T5, 2 h post-surgery.

In the linear mixed-effects model, HPC was associated with significantly improved PaO_2_/FiO_2_ over time, whereas no significant main effect of RIPC or HPC × RIPC interaction was observed. At 30 min of OLV (T2), patients receiving HPC demonstrated significantly higher PaO_2_/FiO_2_ compared with controls [256.4 ± 60.3 vs. 201.3 ± 65.0 mmHg; mean difference (MD) 55.1, 95% CI 12.3–97.9; *p* = 0.022]. The combined HPC + RIPC group showed a comparable improvement [264.1 ± 60.2 mmHg; MD 62.8, 95% CI 23.5–102.1; *p* = 0.007], while RIPC alone did not improve oxygenation [206.1 ± 71.7 vs. 201.3 ± 65.0 mmHg; MD 4.8, 95% CI −9.6–19.2; *p* = 0.992]. These improvements in oxygenation persisted until the termination of OLV (T3) [HPC: 297.5 ± 70.3 vs. Control: 217.8 ± 66.8 mmHg; MD 79.7, 95%CI 42.1–117.3; *p* < 0.001] (Figure 2A). All reported *post hoc p* values were adjusted using the Holm–Bonferroni method.

### 3.3 Secondary outcomes

#### 3.3.1 Ventilation parameters

At T2, HPC was associated with a lower respiratory index (RI) compared with controls [1.8 ± 0.6 vs. 2.6 ± 1.3; MD -0.8, 95% CI, −1.2 to −0.4; *p* = 0.001]. This difference persisted at T3 (1.7 ± 0.6 vs. 2.1 ± 1.3; MD −0.4, 95%CI, −0.8 to −0.02; *p* = 0.04). The alveolar-arterial oxygen gradient (A-aDO2) was also reduced at T2 (237.3 ± 70.5 vs. 294.7 ± 88.7 mmHg; MD −57.4, 95% CI, −101.2 to −13.6; *p* = 0.011) (Figures 2B,C).

#### 3.3.2 Clinical outcomes

HPC was associated with a shorter duration of postoperative mechanical ventilation [median 45 (IQR 30–85) vs. 102.5 (61.3–128.8) minutes; Hodges-Lehmann estimator −57.5, 95% CI −73.2 to −41.8; *p* < 0.001] and a reduced length of hospital stay [7 (5.8–10) vs. 11 (7.5–16.5) days; *p* = 0.001]. The combined HPC + RIPC group showed comparable outcomes [mechanical ventilation duration: 60 (32.5–78.8) minutes; hospital stay: 8.5 (6.0–10) days], with no significant differences compared with HPC alone (both *p* > 0.30) (Table 2).

**Table 2:** Secondary postoperative outcome measures.

Variable	CR (*n* = 37)	HPC (*n* = 34)	RIPC (*n* = 33)	HPC + RIPC (*n* = 35)	*p* value
Extubation time (min)	102.5 (61.3, 128.8)	45* (30, 85)	90 (47.5, 140)	60* (32.5, 78.8)	<0.001*
Length of stay (days)	11 (7.5, 16.5)	7* (5.8, 10)	10 (7, 12.5)	8.5* (6.0, 10)	<0.001*
Total PPCs *n* (%)	30 (81%)	18 (53%)	28 (85%)	23 (65%)	0.014*
Pneumonia	3 (8%)	3 (9%)	5 (15%)	0 (0%)	0.144
Atelectasis	3 (8%)	0 (0%)	3 (9%)	0 (0%)	0.101
Pleural effusion	14 (38%)	7 (21%)	14 (42%)	9 (26%)	0.178
Pneumothorax	6 (16%)	7 (21%)	2 (6%)	3 (9%)	0.188
Subcutaneous emphysema	7 (19%)	9(26%)	9 (27%)	12 (34%)	0.094
ARDS	0 (0%)	0 (0%)	0 (0%)	0 (0%)	–

Data are presented as the count (percentage) and median (IQR), *p < 0.05 versus Control group.

CR, Control; HPC, Hypoxia preconditioning; RIPC, Remote ischemic preconditioning; PPCs, Pulmonary complications; ARDS, Acute respiratory distress syndrome.

#### 3.3.3 PPCs

The incidence of PPCs decreased from 81.1% (30/37) in control group to 52.9% (18/34) in the HPC group (RR 0.65, 95%CI 0.47–0.91; *p* = 0.021). Most PPCs consisted of minor radiographic findings detected during early postoperative surveillance. Subgroup analysis revealed a reductions in pleural effusion (37.8% vs. 20.6%) in the HPC group. RIPC did not reduce PPC incidence (85% vs. 81%; *p* = 0.79) (Table 2).

#### 3.3.4 Inflammatory markers

**Figure 3:**
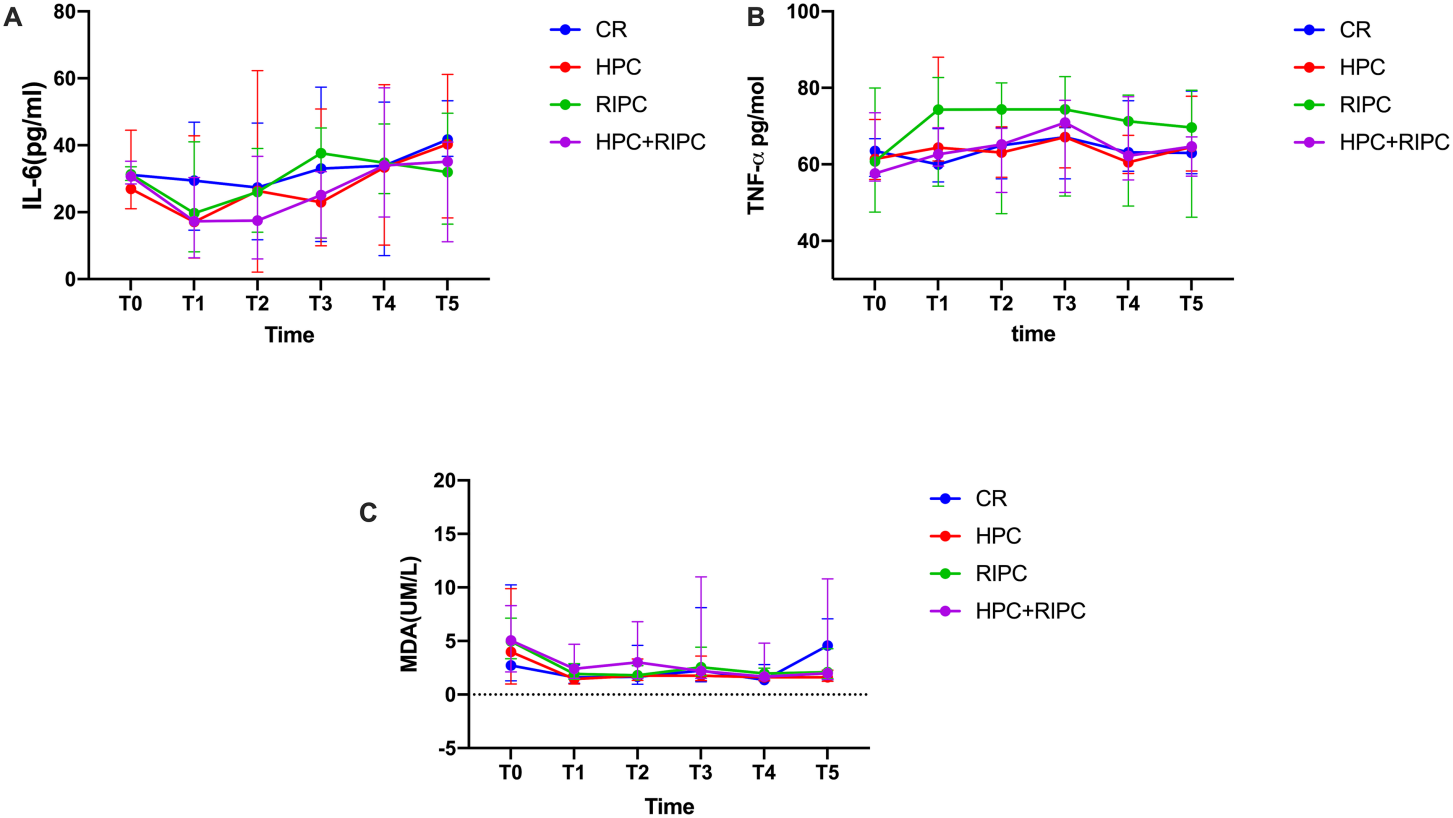
The levels of IL-6, TNF-α, and MDA at a specific period in the four groups. **(A)** Interleukin-6 (IL-6) concentration, **(B)** tumor necrosis factor-α (TNF-α), **(C)** serum malondialdehyde (MDA). Data are presented as mean ± SD. CR, Control; HPC, Hypoxia preconditioning; RIPC, Remote ischemic preconditioning; OLV, One-lung ventilation; T0, after introduction; T1, before OLV; T2, after 30 min of OLV; T3, at the end of OLV; T4, at the end of the surgery; T5, 2 h post-surgery.

No significant intergroup differences emerged in IL-6 trajectories [peak T3: Control (68.4 ± 21.3) vs. HPC (62.1 ± 18.9) pg./mL; MD −6.3, 95%CI, −15.8 to 3.2; *p* = 0.19], TNF-α levels (*p* = 0.34 by Friedman test), or MDA concentrations(*p* = 0.47) (Figure 3).

#### 3.3.5 Safety

All interventions were well tolerated. Hemodynamic parameters remained stable throughout the study, with no clinically significant intergroup differences (Table 3). No device-related complications or unplanned intensive care admissions occurred.

**Table 3:** Hemodynamic and arterial blood gas data.

Variable	CR (*n* = 37)	HPC (*n* = 34)	RIPC (*n* = 33)	HPC + RIPC (*n* = 35)	*p* value
MAP (mmHg)					
T0	59.3 ± 6.6	61.6 ± 7.0	64.1 ± 8.7	64.4 ± 7.2	0.125
T1	62.8 ± 8.4	64.6 ± 10.2	62.5 ± 8.3	62.8 ± 9.2	0.861
T2	56.7 ± 8.7	58.7 ± 7.0	57.2 ± 7.1	58.6 ± 7.4	0.732
T3	57.7 ± 8.2	57.4 ± 6.5	56.0 ± 6.8	56.8 ± 7.3	0.860
T4	58.0 ± 8.6	60.9 ± 8.4	57.1 ± 7.6	58.8 ± 6.9	0.413
T5	60.4 ± 8.1	62.3 ± 6.0	61.2 ± 5.5	60.2 ± 5.5	0.693
HR (bpm)					
T0	107.8 ± 16.9	103.5 ± 12.6	103.2 ± 18.9	102.8 ± 12.8	0.590
T1	104.1 ± 18.6	104.3 ± 14.9	104.1 ± 22.1	102.3 ± 11.8	0.977
T2	105.5 ± 15.4	104.3 ± 13.4	107.6 ± 19.8	104.3 ± 13.0	0.882
T3	106.0 ± 19.2	101.8 ± 12.2	105.1 ± 19.8	100.8 ± 13.2	0.620
T4	104.1 ± 17.7	97.5 ± 14.8	100.3 ± 16.5	99.5 ± 3.0	0.461
T5	106.2 ± 16.4	102.2 ± 12.5	105.2 ± 19.2	101.9 ± 11.8	0.677
Peak^a^ (cmH_2_O)					
T0	17.5 ± 2.8	16.2 ± 3.2	16.5 ± 2.5	16.5 ± 3.4	0.364
T1	19.6 ± 3.2	19.1 ± 4.1	19.6 ± 3.9	18.8 ± 4.1	0.864
T2	20.3 ± 2.3^#^	21.8 ± 3.9^#^	20.2 ± 2.4^#^	21.2 ± 3.5^#^	0.217
T3	19.8 ± 2.9^#^	19.9 ± 4.0^#^	20.6 ± 3.7^#^	20.7 ± 4.4^#^	0.733
T4	17.8 ± 2.6	17.4 ± 3.0	17.6 ± 2.3	18.1 ± 3.4	0.839
SpO_2_ (%)					
T0	100 (100, 100)	100 (99, 100)	100 (100, 100)	100 (100, 100)	0.817
T1	100 (99, 100)	100 (98, 100)	100 (100, 100)	100 (99.3, 100)	0.708
T2	99 (97, 100)	99 (97, 100)	99 (96, 100)	99 (98.3, 100)	0.563
T3	100 (98, 100)	100 (99, 100)	100 (98, 100)	100 (99, 100)	0.321
T4	100 (100, 100)	100 (99, 100)	100 (100, 100)	100 (100, 100)	0.613
T5	100 (100, 100)	100 (100, 100)	100 (99, 100)	100 (99.3, 100)	0.068
PaCO_2_ (mmHg)					
T0	37.5 ± 5.4	35.0 ± 4.1	37.1 ± 9.6	41.3 ± 8.8	0.636
T1	43.9 ± 8.5	42.3 ± 6.1	42.0 ± 5.8	44.7 ± 6.5	0.177
T2	45.5 ± 6.0	45.4 ± 7.8	45.3 ± 8.3	44.7 ± 6.4	0.980
T3	44.7 ± 7.6	43.4 ± 5.8	44.6 ± 7.8	43.2 ± 5.1	0.789
T4	41.8 ± 5.1	42.1 ± 7.1	40.1 ± 3.9	41.0 ± 4.4	0.556
T5	38.7 ± 3.0	41.0 ± 6.0	39.6 ± 6.9	40.1 ± 5.1	0.730
pH					
T0	7.35 ± 0.06	7.37 ± 0.04	7.37 ± 0.05	7.38 ± 0.04	0.134
T1	7.35 ± 0.07	7.35 ± 0.04	7.36 ± 0.05	7.37 ± 0.04	0.338
T2	7.31 ± 0.06	7.33 ± 0.04	7.34 ± 0.04	7.38 ± 0.03	<0.01*
T3	7.33 ± 0.06	7.35 ± 0.05	7.35 ± 0.04	7.35 ± 0.04	0.704
T4	7.34 ± 0.05	7.35 ± 0.05	7.35 ± 0.03	7.35 ± 0.04	0.426
T5	7.37 ± 0.04	7.38 ± 0.04	7.36 ± 0.03	7.37 ± 0.03	0.783
Glu (mmol/L)					
T0	4.9 ± 0.7	5.0 ± 0.7	5.2 ± 0.6	5.1 ± 0.7	0.443
T1	4.9 ± 0.8	4.9 ± 0.8	5.0 ± 0.5	4.9 ± 0.7	0.892
T2	5.4 ± 0.9	5.3 ± 0.6	5.5 ± 0.8	5.5 ± 0.8	0.813
T3	5.4 ± 0.9	5.7 ± 1.0	5.7 ± 0.7	5.7 ± 0.9	0.349
T4	5.6 ± 0.9	5.6 ± 0.8	5.6 ± 0.9	5.7 ± 0.9	0.957
T5	5.4 ± 0.7	6.0 ± 0.9	5.7 ± 0.8	5.9 ± 1.1	0.086
Hb (g/dl)					
T0	10.2 ± 1.3	10.5 ± 1.1	9.9 ± 1.4	10.4 ± 0.9	0.424
T1	9.9 ± 1.3	10.2 ± 1.2	9.7 ± 1.3	10.0 ± 1.1	0.590
T2	9.8 ± 1.3	10.2 ± 1.1	9.9 ± 1.5	10.1 ± 1.1	0.624
T3	9.7 ± 1.1	9.9 ± 1.0	10.1 ± 1.3	10.1 ± 1.2	0.579
T4	9.7 ± 0.9	9.7 ± 1.0	10.1 ± 1.0	10.1 ± 1.3	0.513
T5	10.4 ± 1.1	10.9 ± 1.1	10.5 ± 1.0	10.9 ± 1.2	0.215

Data are presented as mean±standard deviation or median (IQR).

*p < 0.05 versus control group, ^#^*p* < 0.05 versus T0.

a Peak values were not recorded at T5 due to early extubation.

MAP, mean arterial pressure; HR, heart rate; SPO_2_; PCO_2_; PH; Glu; Hb, hemoglobin; CR, Control; HPC, Hypoxia preconditioning; RIPC, Remote ischemic preconditioning; OLV, One-lung ventilation; T0, after introduction; T1, before OLV; T2, after 30 min of OLV; T3, at the end of OLV; T4, at the end of the surgery; T5, 2 h post-surgery.

## 4 Discussion

Our randomized trial demonstrates that HPC is associated with improved intraoperative oxygenation and enhanced early postoperative recovery in children undergoing thoracoscopic surgery with OLV. Three principal findings emerge from our study: (1) HPC significantly improves intraoperative PaO_2/_FiO_2_ during OLV, with effects persisting until the end of OLV; (2) These physiological improvements were accompanied by shorter postoperative mechanical ventilation duration and reduced hospital length of stay; (3) HPC was associated with a lower incidence of PPCs. In contrast, RIPC, either alone or combined with HPC, did not confer additional benefit in this pediatric cohort.

These findings are consistent with experimental and clinical studies suggesting that HPC may exert lung-protective effects under hypoxic conditions (16–19). In preclinical models, these effects have been linked to optimization of hypoxic pulmonary vasoconstriction (HPV) and stabilization of pulmonary microvasculature. While our study did not directly assess shunt fraction, pulmonary vascular resistance, or HPV responsiveness, the observed improvement in PaO_2/_FiO_2_ during OLV is compatible with enhanced ventilation-perfusion matching, a hallmark consequence of effective HPV.

Notably, the magnitude of oxygenation improvement observed in our pediatric cohort exceeded that reported in adult trials (9). This difference may reflect age—related physiological factors, including heightened hypoxic responsiveness of the developing pulmonary vasculature. Prior studies have shown that intermittent hypoxic stimuli can augment HPV and reduce intrapulmonary shunt during subsequent OLV (20). Given that pediatric lungs exhibit greater vascular reactivity to hypoxia (21), such mechanisms may be more pronounced in children; however, this interpretation remains hypothesis-generating and warrants direct physiological investigation.

The temporal pattern of oxygenation improvement—emerging early during OLV and persisting thereafter—further supports a role for acute physiological adaptation rather than delayed systemic effects. With respect to clinical outcomes, although the incidence of PPCs in the HPC group remained relatively high, it was substantially lower than in the control group. Overall PPC rates in our cohort appear higher than those commonly reported in adult thoracic surgery cohorts (22). This elevated baseline PPC rate likely reflects anatomical characteristics of pediatric airways and the protocol—mandated universal chest tube placement, which has been associated with increased postoperative pleural complications in pediatric thoracic surgery (23). Importantly, the reduction in pleural effusion observed with HPC is consistent with prior experimental evidence suggesting hypoxia—induced resistance to microvascular leakage (19), indicating potential benefits beyond gas exchange alone.

Analysis of inflammatory and oxidative stress biomarkers (IL-6, TNF-α, and MDA) did not reveal significant intergroup differences. This contrasts with some adult studies in which systemic inflammatory modulation has been proposed as a key mechanism of preconditioning (10). However, mechanistic human studies have also suggested that RIPC does not consistently attenuate systemic inflammatory responses *in vivo* (24). Our findings suggest that, in pediatric thoracoscopic surgery, the beneficial effects of HPC may be predominantly mediated through localized pulmonary physiological mechanisms rather than systemic anti-inflammatory pathways. Nevertheless, this interpretation should be considered exploratory given the absence of direct mechanistic measurements.

In contrast to reports of RIPC benefits in adult thoracic and cardiac surgery (25, 26), we observed no protective effects of RIPC in this study. Several factors may account for this discrepancy. Routine administration of dexamethasone and continuous propofol infusion—both standard components of pediatric anesthesia—may attenuate cytokine—or reactive oxygen species—dependent signaling pathways implicated in RIPC (26, 27). Additionally, OLV-related lung injury may differ fundamentally from classical ischemia—reperfusion models in which RIPC has demonstrated protective effects, as the pathophysiology of OLV injury involves ventilation—induced and inflammatory mechanisms rather than pure ischemia—reperfusion injury (2, 27). Our findings are consistent with recent pediatric and neonatal studies reporting limited or context-dependent benefits of RIPC (13). Although recent meta-analyses have suggested that RIPC may improve certain clinical outcomes in noncardiac surgery, these effects appear to vary substantially across surgical populations and perioperative conditions (28).

### 4.1 Study limitations

Several limitations should be acknowledged. First, the single-center design and sample size may limit generalizability, although our institution is a high-volume tertiary pediatric center. Second, universal chest tube placement may have increased the baseline incidence of PPCs relative to centers employing selective drainage strategies. Third, standardized anesthetic regimens incorporating propofol and dexamethasone—while reflective of real-world practice—may have obscured potential RIPC effects.

Finally, we acknowledge a deviation from the initial trial registration with respect to mechanistic biomarkers. Although NF-κB, IL-10, and SOD were listed as planned indicators, these markers were not analyzed due to logistical and technical constraints affecting sample integrity. Importantly, the primary clinical endpoint and statistical analysis plan were prospectively defined prior to data analysis, and the observed clinical benefits of HPC should be interpreted independently of these exploratory molecular measures.

## 5 Conclusion

In summary, this randomized trial demonstrates that HPC is associated with improved intraoperative oxygenation and accelerated postoperative recovery in children undergoing thoracoscopic surgery with one lung ventilation. In contrast, RIPC, alone or in combination, provided no additional pulmonary protective benefits in this specific pediatric cohort. Our findings suggest that HPC may exert its protective effects primarily through acute physiological optimization—likely via modulation of hypoxic pulmonary vasoconstriction—rather than through systemic anti-inflammatory pathways. Given its non-invasive nature, favorable safety profile, and association with reduced hospital length of stay, HPC warrants further investigation and consideration as a lung-protective strategy in pediatric thoracic anesthesia.

## Data Availability

The raw data supporting the conclusions of this article will be made available by the authors, without undue reservation.
